# Environmental impacts due to the use of sunscreen products: a mini-review

**DOI:** 10.1007/s10646-022-02592-w

**Published:** 2022-09-29

**Authors:** Myrto Chatzigianni, Panagoula Pavlou, Angeliki Siamidi, Marilena Vlachou, Athanasia Varvaresou, Spyridon Papageorgiou

**Affiliations:** 1 Department of Biomedical Sciences, Division of Aesthetics and Cosmetic Science, School of Health and Care Sciences, University of West Attica, 28 Ag. Spyridonos Str., 12243 Egaleo, Greece; 2 Laboratory of Chemistry-Biochemistry-Cosmetic Science, Department of Biomedical Sciences, School of Health and Care Sciences, University of West Attica, 28 Ag. Spyridonos Str., 12243 Egaleo, Greece; 3 Department of Pharmacy, Division of Pharmaceutical Technology, School of Health Sciences, National and Kapodistrian University of Athens, 15784 Athens, Greece

**Keywords:** Sunscreen filters, Marine environment, Bioaccumulation, Biomagnification, Marine biocommunities, Ecotoxicology

## Abstract

Sunscreen use has increased in recent years, as sunscreen products minimize the damaging effects of solar radiation. Active ingredients called ultraviolet (UV) filters or UV agents, either organic or inorganic, responsible for defending skin tissue against harmful UV rays, are incorporated in sunscreen formulations. UV agents have a serious impact on many members of bio communities, and they are transferred to the environment either directly or indirectly. Many organic UV filters are found to be accumulated in marine environments because of high values of the octanol/water partition coefficient. However, due to the fact that UV agents are not stable in water, unwanted by-products may be formed. Experimental studies or field observations have shown that organic UV filters tend to bioaccumulate in various aquatic animals, such as corals, algae, arthropods, mollusks, echinoderms, marine vertebrates. This review was conducted in order to understand the effects of UV agents on both the environment and marine biota. In vivo and in vitro studies of UV filters show a wide range of adverse effects on the environment and exposed organisms. Coral bleaching receives considerable attention, but the scientific data identify potential toxicities of endocrine, neurologic, neoplastic and developmental pathways. However, more controlled environmental studies and long-term human use data are limited. Several jurisdictions have prohibited specific UV filters, but this does not adequately address the dichotomy of the benefits of photoprotection vs lack of eco-friendly, safe, and approved alternatives.

## Introduction

Sunscreens have been the cornerstone of sun protection, since they were introduced to the public in 1928 (Sambandan and Ratner 2011). They minimize the damaging effects of solar radiation, such as premature ageing of the skin, erythema and sunburn. Sunscreen formulations include certain active ingredients, called ultraviolet (UV) filters, which could either be organic or inorganic, generally known as “chemical” and “physical”, respectively. These ingredients are responsible for defending skin tissue against harmful UV rays (Tsirivas et al. 2016). In many cases, the available formulae may contain a combination of both kinds of filters, in order to offer sun protection on a broad spectrum (Stiefel and Schwack 2015).

Organic UV agents act by absorbing UV radiation and converting it to harmless heat energy, which is released at a later stage (Sambandan and Ratner 2011). When the molecules of these agents are exposed to UV radiation, their electrons receive energy and tend to get excited. In their neutral state, the same energy is converted to thermal energy (Tsirivas et al. 2016). For this reason, consumers may feel a slight change of temperature when applying sunscreen products under the sun (Draelos 2006). UV agents of this category are divided into UVA and UVB filters, depending on their absorption properties. Most organic filters are aromatic compounds, conjugated with carbonyl groups. The electrons of the benzene ring are the ones that get excited when the absorption of UV radiation takes place (Stiefel and Schwack 2015). Several examples of known organic UV agents are dibenzoylmethane, benzophenone, salicylate, cinnamate and camphor derivatives, *p*-aminobenzoic acid, triazones and triazines (Sambandan and Ratner 2011, Stiefel and Schwack 2015). Despite their safety and stability, some organic UV filters are known to cause negative cutaneous effects, such as dermatitis and eczema, and have been detected in human urine, blood and even breast milk (Janjua et al. 2008, Matta et al. 2020, Ngoc et al. 2019, Pavlou et al. 2021).

Inorganic sunscreen agents are able to reflect, scatter and partly absorb sunrays; combined, these properties offer protection across both the UVA and the UVB spectrum (Stiefel and Schwack 2015). The effectiveness of such ingredients depends on several variables, such as particle size, dispersion of particles in the emulsion, amount of sunscreen product used, and index of refraction of each UV agent (Tsirivas et al. 2016). The most commonly known filters in this category are titanium dioxide (TiO_2_) and zinc oxide (ZnO). They are considered to be less toxic and safer for human use due to the fact that they do not penetrate deep into the skin (Ngoc et al. 2019, Palm and O’Donoghue 2007). In the 1980s, TiO_2_ and ZnO were incorporated into sunscreen formulae in the form of larger particles (Schneider and Lim 2019). TiO_2_, which is mainly used in cosmetics, exists in three crystalline forms: anatase, brookite and rutile (Manaia et al. 2013, Stiefel and Schwack 2015). It offers protection against the UVB range, but lacks coverage within the UVA region, whereas ZnO is effective against a wide UV range (Palm and O’Donoghue 2007). Nowadays, mostly nano-sized particles are incorporated in sunscreen formulations; nano-sized particles or “nanoparticles” refer to particles which are smaller than 100 nm in diameter. This distinction is important because TiO_2_ and ZnO particles range in size from 150–300 nm and 200–400 nm, respectively (Schneider and Lim 2019). Incorporation of UV filters in the form of nanoparticles in sunscreen formulas leads to better radiation absorption and enhanced overall appearance and performance (Manaia et al. 2013). However, in order to include inorganic UV agents in sunscreen products, surface coating is being used, since the small particles of the former tend to form aggregates. Coating is also used to achieve elimination of the photocatalytic properties of TiO_2_. Inorganic layers like aluminium oxide Al_2_O_3_, aluminium hydroxide Al(OH)_2_ and hydrated silica SiO_2_ are chosen to passivate the TiO_2_ surface (Heilgeist et al. 2021).

Both organic and inorganic filters have been detected in marine ecosystems through water sampling from various places around the globe. The concentration of pollutants varies and depends on several factors, such as the area, date and time at which the sampling takes place and whether it is visited by swimmers (Giokas et al. 2007). Reportedly, the highest concentrations of UV filters are detected in waters close to frequently visited beaches such as Hawaii, Okinawa Island, Majorca Island, Virgin Islands, the Southern Baltic Sea, the Mediterranean Sea (Astel et al. 2020, Bargar et al. 2015, Tashiro and Kameda 2013, Tovar-Sánchez et al. 2013). The concentrations of 13 UV-filters were measured in surface seawater, sediment, and coral tissue from 19 sites in Oahu, Hawaii. At least eight UV-filters were detected in seawater with concentrations lower than 750 ng L^−1^. These UV-filter concentrations generally varied as follows: water, homosalate (HMS) > octisalate (OS) > benzophenone-3 (BP-3)>octocrylene (OC); sediment, HMS > OS > OC > BP-3; coral, OS ≈ HMS > OC ≈ BP-3 (Mitchelmore et al. 2019).

In this article we review the information derived from the bibliographical data regarding the biochemical influence of the inorganic and organic sunscreens on organisms of the aqueous environment. The consequences of the intervention of sunscreens on the metabolism of corals, algae, arthropods, mollusks, echinoderms are described. Moreover, the transportation of the sunscreens to other aqueous non touristic ecosystems via the phenomenon of biomagnification seems also worrying. Additionally, we list regulatory actions that have been taken by some States and countries regarding the use of specific sunscreens, although the number of long-term controlled and real-time environmental studies is low.

## Pathways of UV filters from sunscreens to the environment

UV agents are transferred to the environment either directly or indirectly (Giokas et al. 2007). Tourism plays a major role in the direct transfer of UV filters to aqueous environments. Coastal tourism is constantly increasing as a growing number of people spend their time by the sea. Sánchez-Quiles and Tovar-Sánchez (2015) estimated that the total number of tourists has increased by 65% to 763 million people in 2004 compared to 1992. Moreover, the number of inhabitants in coastal areas is expected to increase to 5.2 billion by 2080, in comparison to 1.2 billion in 1990 (Rabalais et al. 2009). Many new recreational activities have been developed in order to satisfy tourists’ needs, such as various water sports, fishing etc. It has been reported that the increase of tourist influx in coastal areas and concentration of UV agents in waters of these areas are correlated (Sánchez-Quiles and Tovar-Sánchez 2015, Tovar-Sánchez et al. 2019). However, apart from sunscreens, these active ingredients are included in other cosmetic products, which can also contribute to marine pollution; moisturizers, shampoos and makeup products in general, may contain UV filters as well (Chisvert et al. 2013). It is estimated that 25% of the applied sunscreen is deposited to water (Danovaro et al. 2008).

Indirect transfer of UV filters to ecosystems is attributed to wastewater systems. Showering, urinating and washing clothes are actions that allow sunscreen residue to end up in waters, which is later present in wastewater treatment plants (WWTP), if these are available. During this process, the pollutants are not efficiently removed, so effluents with residual UV agents are transferred to marine ecosystems and even to tap water (Sánchez-Quiles and Tovar-Sánchez 2015). Additionally, in regions where WWTPS are not available, which is the case for many island states, UV filters and other cosmetic products are transferred to the marine environment through the sewage system (Amine et al. 2012).

The photostability of UV agents is desirable when applied to the skin in sunscreen formulations, whereas in aquatic environments their effect is lessened, and formation of harmful by-products may take place. This happens due to the process of direct or indirect photolysis. During direct photolysis, filters are decomposed via reactions triggered by solar energy. On the other hand, in indirect photolysis other factors contribute to filter decomposition, such as reactive oxygen species (ROS) and inorganic compounds (Sánchez-Quiles and Tovar-Sánchez 2015). Instances of such injurious by-products are substituted benzoic acids, dibenzoylmethanes, 4-methoxybenzaldehyde, cyclodimers (Santos et al. 2012). It is important to note that photolysis is perilous in chlorous environments, for example pool water, because the chlorinated by-products present pose a threat to human health (Manasfi et al. 2017). The degradation of the covering in TiO_2_ nanoparticles may result in the formation of ROS (Slomberg et al. 2021). In general, these nanoparticles have a tendency to form agglomerates, especially when combined with other materials. These newly formed particles can be toxic to biota (Abdel-Latif et al. 2020).

Most organic UV filters tend to bioaccumulate in biota due to the high values of octanol/water partition coefficient (Kow) (Sánchez-Quiles and Tovar-Sánchez 2015). This coefficient indicates the relationship between the fat and water solubility of a substance; if its value is greater than 1 (Kow>1), then the substance is easily soluble in fatty solvents, whereas if its value is lower than 1 (Kow < 1), it is considered as soluble in water. Consequently, organic UV filters have been reported to bioaccumulate in various aquatic animals. However, these pollutants are not limited to marine environments only, since they have been detected in Swiss cormorants and bird eggs in Spain (Fent et al. 2010, Molins-Delgado et al. 2017). This fact illustrates the severity of biomagnification, a phenomenon which is very important in the ecosystem pollution analysis. Exposure to sunscreens impacts three basic functions of animal life: survival, development and reproduction (Tovar-Sánchez et al. 2019). The endocrine system can specifically be affected (Fent et al. 2010, Tovar-Sánchez et al. 2019). Negative effects of UV filters on corals, algae, arthropods, mollusks, echinoderms, vertebrates are presented and analyzed below.

## Effects on corals

Human stressors including water pollution, tourism, overfishing, destructive fishing, more severe storms owing to climate change, etc. are to blame for the dramatic loss of corals worldwide (Williams et al. 2019). As for the effects of sunscreens on corals, certain active ingredients can cause bleaching, which eventually might lead to their death (Spalding et al. 2001). In many cases bleaching can be reversible, however unless the exposure to negative phenomena is short, coral death is imminent (Baker et al. 2008, Hoegh-Guldberg et al. 2019, Spalding et al. 2001). Coral reefs are also sensitive to environmental changes (Raffa et al. 2019); fluctuations of water salinity levels, temperature and pH may bring about various issues to corals, primarily coral bleaching (Mahmoud et al. 2016).

Certain dinoflagellates endosymbionts (“zooxanthellae”) power the growth of stony corals and coral reef ecosystems (Lesser 2006). Once assumed to encompass a single panmictic species, genetic evidence has revealed a rich diversity within the zooxanthella genus Symbiodinium. With the consideration of molecular, morphological, physiological, and ecological data, proposed that evolutionarily divergent Symbiodinium “clades” are equivalent to genera in the family Symbiodiniaceae, and provided formal descriptions for seven of them.

Many UV filters, as octocrylene, benzophenone, camphor derivatives, ethylhexyl methoxycinnamate, are shown to cause coral bleaching (Table 1); the reason for this being the fact that these filters have negative effects on zooxanthellae (Danovaro et al. 2008). For example, BP-3 can be toxic to corals causing tissue necrosis, which is caused by ROS formation. ROS can also affect the photosynthetic mechanism within the zooxanthellae (Danovaro et al. 2008, Wijgerde et al. 2020). However, Danovaro et al. (2008) did not monitor any water quality parameters such as pH or oxygen levels, even though they are important for coral health (Moeller et al. 2021). Regarding inorganic sunscreen agents, both TiO_2_ and ZnO nanoparticles can pose negative impacts to corals (see Table 1). The latter is known to be fatal to dinoflagellates when the nanoparticles are not covered. To elaborate, a study concluded that coral exposure to nanoparticles of such nature leads to zooxanthellae loss within the tissues and, eventually, to bleaching of the *Acropora* coral (Danovaro et al. 2008, Corinaldesi et al. 2018). It is worth mentioning that Corinaldesi et al. (2018) found that exposure of corals to coated TiO_2_ nanoparticles caused no bleaching and minimal systemic changes between zooxanthellae and the corals themselves. In another experiment, 17-day exposure to titanium dioxide nanoparticles was the cause of slight bleaching and zooxanthellae expulsion (zooxanthellae density was 14 and 25% depending on the treatment concentration) in the *Montastraea faveolata* species (Jovanović and Guzmán 2014). Since control groups with larger sized TIO_2_ particles were missing in this study, it is not clear whether the effects observed, were caused by intrinsic properties of TIO_2_, a nano-specific response, or solely by a particle effect of the test material (Moeller et al. 2021, Heilgeist et al. 2021).

**Table 1:** Effects of UV filters on corals

UV Filter	Effect on Corals	Coral Species Studied	Test Concentration Ranges	Toxicity Threshold	Duration of Study	Reference
OXYBENZONE	Negative effect on the photosynthetic mechanisms of zooxanthellae due to ROS	*Stylophora pistillata, Acropora tenuis*	C = 0.06 μg/L	na	6 weeks	Wijgerde et al. 2020
EHMC	Mortality, Visual bleaching	*Seriatopora caliendrum, Pocillopora damicornis*	C = 33.5 ± 7.6 μg/L	LOEC: 1 mg/L (1000 μg/L)	7 days	He et al. 2019
ZnO (nano)	Death of zooxanthellae, Enrichment of viruses and prokaryotes around corals, Bleaching	*Acropora spp*.	C = 6.3 mg/L	na	48 h	Corinaldesi et al. 2018
TiO_2_ (nano)	Elimination of zooxanthellae	*Montastraea faveolata*	C = 10 mg/L	na	17 days	Jovanović and Guzmán 2014
BENZOPHENONE-3	Interspecific variations in toxicity	*Leptastrea purpurea, Tubastraea faulkneri, Acropora digitifera, A. millepora*	C = 83.3 to 1.3 μg/L *(L. purpurea, A. Digitifera)* C = 5.3 mg/L to 1.3 μg/L *(A. millepora)* C = 5.3 mg/L to 166.7 μg/L *(T. faulkneri)*	EC_50_ = 1.84 µg L^−1^ (*Leptastrea purpurea)* LC_50_ = 0.75 µg L^−1^ *(A. digitifera)* LC_50_ = 2951.24 µg L^−1^ *(T. faulkneri)*	48 h under static conditions	Miller et al. 2022

Coral reefs have been declining globally at a historically unprecedented rate. UV filters used in sunscreens may contribute to this decline at local scales, which has already led to bans on various organic UV filters in some regions (e.g., Hawaii, Palau) (Miller et al. 2021). However, the underlying studies for these bans demonstrated significant flaws in the experimental design due to a lack of validated and standardized testing methods for corals (e.g., OECD, DIN, ISO) (Mitchelmore et al. 2019, Moeller et al. 2021, Burns et al. 2021). Recently, Miller et al. (2022) suggested a reliable bioassay to measure the acute reactions of planula larvae of four species of scleractinian coral to widely used BP3. In assessing coral larvae toxicity, it has been discovered that mortality and settlement (metamorphosis and attachment) are effective outcomes. For the four investigated species, there were interspecific variances in the toxicity thresholds based on measured exposure amounts, ranging from low µg L^−1^ to low mg L^−1^. With an EC50 of 1.84 µg L^−1^, settlement inhibition has been occured in larvae taken from the brooding coral *L. purpurea*. The results of the BP3 experiments revealed that the test system with coral larvae, the test duration of 48 h and the investigated endpoints, settlement and mortality, are suitable for further validation and international standardization within the ISO and/or OECD framework.

## Effects on algae

Algae are vital eukaryotic organisms that have been living on the planet Earth for almost 3.5 billion years (Chapman 2013). Thanks to their photosynthetic abilities, they are able to produce very important compounds, as well as their own food, since they are classified as autotrophic organisms. Algal products include proteins, carbohydrates, antioxidants, pigments and fatty acids (Michalak and Chojnacka 2015). They also enrich the oxygen of the atmosphere through photosynthesis; at least 50% of all oxygen production is attributed to phytoplankton (Chapman 2013). They are prominent in both marine and terrestrial ecosystems (Spalding et al. 2001, Wang et al. 2015). Algae are mostly found in surface water and are resistant to environmental changes in temperature, water salinity and pressure (Christaki et al. 2013, Pereira 2021).

Exposure to iron oxide nanoparticles is a great threat to algae. Concerning TiO_2_ nanoparticles, when they are exposed to ultraviolet radiation, ROS formation is observed. ROS can cause oxidative stress to algae, as they are absorbed to the outer layer of phytoplankton and oxidative reactions on the cell walls take place. Additionally, ROS can cause molecular changes to cellular membrane lipids and proteins. They can also inhibit the production of photosynthetic pigments, such as chlorophyll, and as a result, affect the process of photosynthesis (Dalai et al. 2013, Miller et al. 2012). Uncoated ZnO nanoparticles can hinder the development of algae when combined with exposure to UVA and UVB radiation, as concluded by experiments on the species *Pseudokirchneriella subcapitata* by Lee and An (2013) and *Chlorella sp*. (Ji et al. 2011). Zinc ions (Zn^2+^) can also be toxic to algae, as cellular membrane, mitochondria and DNA damage is attributed to them (Franklin et al. 2007, Lee and An 2013).

As for organic UV agents, the *p*-aminobenzoic acid derivative OD-PABA has been proven to be harmful to *Isochrysis galbana* (Giraldo et al. 2017). Gradual increase of oxybenzone levels have also been found to impede development of *Chlamydomonas reinhardtii* and *Chlorella UMACC 401*, whereas *Scenedesmus quadricauda* showed resistance to the compound, apart from the fact that increase of its cellular size was noted (Teoh et al. 2020). In another study, oxybenzone has been shown to be able to decrease production levels of chlorophyll-a in *Chlamydomonas reinhardtii* (Mao et al. 2017).

## Effects on arthropods

Nanoparticles pose a great threat to arthropods, as well. Experimental studies on the species *Tigriopus japonicus* showed that nano-sized ZnO can trigger oxidative stress. Furthermore, 4-day exposure to the inorganic UV agent led to an increase antioxidants’ production within the crustacean (Wong et al. 2020). In another study, embryonic mortality of the same species was noted after exposure to 4-methylbenzylidene camphor (4-MBC), a camphor derivative, along with the same results on antioxidants as the previous analysis (Chen et al. 2018) (see Table 2). Larval growth of *Chironomus riparius* was decreased after exposure to the same camphor product, a phenomenon which can later influence arthropod fertility and reproduction. Exposure to octocrylene and oxybenzone had the same effect (Campos et al. 2017). Additionally, the latter can trigger activation of stress gene hsp70 and other genes related to ecdysis of *Chironomus riparius* (Ozáez et al. 2014).

**Table 2:** Effects of UV filters on arthropods

UV Filter	Effects on Arthropods	Arthropod Species Studied	Test Concentration Ranges	Toxicity Threshold	Duration of Study	Reference
4-MBC	Lethal toxicity of nauplii	*Tigriopus japonicus*	C = 0 to10 μg/L	LC_50_ = 8.5 μg/L	72 h	Chen et al. 2018
	Reduction of reproduction and body length	*Tigriopus japonicus*	C = 0 to10 μg/L	LOEC = 50 μg/L		Chen et al. 2018
3-BC	Irregularities in hepatopancreatic cells	*Gammarus fossarum*	C = 300 μg/L	na	4 days	Scheil et al. 2008
	Changes in protein concentration Hsp70	*Gammarus fossarum*	Maximum response at C = 3.3 μg/L	na		Scheil et al. 2008
AVOBENZONE	Increase in reproduction rate	*Daphnia magna*	C = 20 μg/L	na	21 days	Boyd et al. 2021
OCTOCRYLENE	Effect on the motor responses to light stimuli	*Daphnia magna*	C = 200 μg/L	na	48 h	Boyd et al. 2021

The levels of the hsp70 protein have been found to be affected by exposure to 3-benzylidene camphor (3-BC) in the crustacean *Gammarus fossarum*. Increase of this protein is synonymous with oxidative stress taking place in the organism. However, in this particular study, production of this protein was drastically decreased after a while, which means the animal was not able to confront stress-inducing factors. Deformities of tissues were also detected, which, in the long run, can hinder food digestion (Scheil et al. 2008).

2,4-Dihydroxybenzophenone (Benzophenone-1) is also damaging to arthropods, as seen in a study performed on *Acartia tonsa*. A correlation between environmental salinity or temperature levels and toxicity of this UV agent was noted. In such cases, metabolic rates of organisms are higher, so copepods have to expend twice the metabolic energy expected, leading to faster energy depletion, and as a result, vulnerability to toxic substances in the environment (Kusk et al. 2011).

*Daphnia magna* is a crustacean often used in studies, due to its responsive nature to environmental changes (Altshuler et al. 2011). Given this fact, there is a plethora of data regarding the effects of various sunscreen agents on this arthropod. For example, avobenzone and octocrylene are proven to be toxic to *Daphnia magna*; the latter even in environmentally realistic concentrations. Both UV agents can temporarily interfere with the phototactic abilities of the organism. Avobenzone can also change the reproduction rate of this crustacean. Furthermore, development is known to be affected by both 4-MBC and EHMC (Boyd et al. 2021, Sieratowicz et al. 2011). The toxicity of oxybenzone to this species has also been researched by Du et al. (2017).

## Effects on mollusks

Generally, bivalves (located in beaches, big rocks, and the ocean floor) experience the phenomenon of metal bioaccumulation, which depends on the size of these metal particles. It has been reported that particles over 5 μm can enter the shell, including aggregates of nanoparticles formed in water. These particles may be ingested and transferred to the digestive system (Doyle et al. 2015). From that point, they might end up in the hemolymph, which is responsible for many systemic changes, such as production of various compounds that protect the animal. This phenomenon cannot be blamed in and of itself for causing death to bivalves; however, it can affect important activities, such as food intake (Canesi and Corsi 2016).

Inorganic sunscreen agents can be harmful to abalones, as they can potentially cause oxidative stress. In a related study, after a 4-day exposure to uncoated TiO_2_ nanoparticles, changes in the levels of antioxidants were noted in the species *Haliotis diversicolor supertexta*. To elaborate, the levels of superoxide dismutase were increased, whereas those of glutathione were decreased (see Table 3). These differences in concentrations show that the animal suffered from oxidative stress, something that is corroborated by the production of ROS and nitrogen monoxide (NO) inside the organism (Zhu et al. 2011).

**Table 3:** Effects of UV filters on mollusks

UV Filter	Effects on Mollusks	Mollusk Species Studied	Test Concentration Ranges	Toxicity Threshold	Duration of Study	Reference
TiO_2_ (nano)	Changes in antioxidant enzyme levels	*Haliotis diversicolor supertexta*	C = 0.1 mg/L, C = 1 mg/L, C = 10 mg/L	LOEC = 1.0 mg/L and C ≥ 1.0 mg/L	96 h	Zhu et al. 2011
	Changes in metallotheionin levels	*Mytillus galloprovincialis*	C = 2.8 μg/L, C = 28 μg/L, C = 280 μg/L	LOEC = 2.8 μg/L	24 h	Sureda et al. 2018
	Reduction of byssal secretion	*Mytilus coruscus*	C = 100 μg/L	na	10 days	Shi et al. 2020
ZnO (nano)	Reduction of byssal secretion	*Mytilus coruscus*	C = 100 μg/L	na	10 days	Shi et al. 2020

Similarly, when studying the scallop species *Chlamys farreri*, superoxide dismutase catalase and acetylcholinesterase concentrations were increased after subjects were exposed to TiO_2_. The latter enzyme is linked to neurotoxicity, while the first two indicate oxidative stress taking place inside the organisms. Changes to the gills and the digestive system were also present. The authors of this study did not mention whether the nanoparticles used were coated or not (Xia et al. 2017). Both uncoated TiO_2_ nanoparticles and coated, had a similar effect on the clam *Ruditapes decussates*, causing a rise of antioxidant enzymes (Saidani et al. 2019).

Concerning the mussel, the species *Mytilus galloprovincialis* found in the Mediterranean Sea has been included numerous times in studies related to sunscreen effects on marine life. Mussels are able to produce metallothioneins, which are proteins that protect the animal from the toxicity by metals. Research has shown that exposure to uncoated inorganic UV agents, particularly TiO_2_, can lead to increase of these proteins (Sureda et al. 2018). Another fact about mussels is their tendency to attach to surfaces or even other mussels, so as to gain stability and safety. This is possible due to byssus, a bundle of proteinacious filaments secreted by the animal, which are really sensitive to environmental changes. Absence of the byssus or even decrease of its production is especially dangerous for mussels, as survival or reproduction is at stake in such cases. Exposure of *Mytilus coruscus* to uncoated nano-sized TiO_2_ and ZnO caused a decline of byssus production, supposedly due to energy depletion generated by these metal oxides (Shi et al. 2020) (see Table 3).

Organic UV agents are also prevalent in bivalves. Research has indicated that EHMC and octocrylene have been detected in mussels, such as *Mytilus galloprovincialis* and *Mytilus edulis*, near French coasts, especially during the summer months, when tourism flourishes (Bachelot et al. 2012).

Cephalopods are also prone to experiencing abnormalities caused by sun products. A study on the species *Octopus vulgaris* demonstrated that injection of TiO_2_ led to a temporary increase of NO concentrations in the hemolymph, and of lysozyme, an enzyme related to risks such as infections (Grimaldi et al. 2013).

## Effects on echinoderms

Inorganic UV filters can inhibit the correct development of echinoderm larvae. In a study on the urchin species *Strongylocentrotus purpuratus*, growth abnormalities were observed on larvae after exposure to ZnO, even on concentrations as low as C = 0.001 ppm. These structural abnormalities ranged frοm urchins having shorter limbs, being smaller in general, lacking symmetry to having no skeletal development whatsoever. These results stem from the effect zinc has on calcium carbonate; in aquatic environments, zinc ions (Zn^+2^) may replace calcium ions (Ca^+2^) and reduce skeletal calcification (Cunningham et al. 2020). In another study, TiO_2_ brought about changes to the acetylcholinesterase levels in the species *Paracentrotus lividus* (Gambardella et al. 2013), which happened even after exposure to “eco-friendly” sunscreen that lacked oxybenzone, homosalate and nano-sized TiO_2_ (Corinaldesi et al. 2017, Catalano et al. 2020, Moeller et al. 2021) (see Table 4).

**Table 4:** Effects of UV filters on echinoderms

UV Filter	Effects on Echinoderms	Echinoderm Species Studied	Test Concentration Ranges	Toxicity Threshold	Duration of Study	Reference
ZnO	Skeletal abnormalities	*Strongylocentrotus purpuratus*	C = 0.01 mg/L (ppm)	na	96 hpf	Cunningham et al. 2020
TiO_2_ (nano)	Anomalous development of embryos	*Paracentrotus lividus*	C = 0.001 mg/L, C = 0.01 mg/L, C = 0.1 mg/L, C = 1 mg/L	LOEC = 0.01 mg/L	24 h, 48 h and 72 h	Gambardella et al. 2013
	Changes in cholinesterase concentrations	*Paracentrotus lividus*	C = 0.001 mg/L, C = 0.01 mg/L, C = 0.1 mg/L, C = 1 mg/L	LOEC = 0.001 mg/L		Gambardella et al. 2013

## Effects on marine vertebrates

Fish are often used as objects of studies related to the effects of sunscreens on marine biota. The *Danio rerio* species (also known as zebrafish) is a commonly studied object. When oxybenzone is present in high concentrations (1000 µg/L), expression of the VTG1 (vitellogenin 1) gene is triggered, and consequently, the VTG protein is produced in the liver (Rodríguez-Fuentes et al. 2015). This polypeptide is the precursor of the egg yolk, produced in the female liver, and male fish cannot synthesize it, unless exposed to exogenous estrogen. High concentrations of VTG are related to toxic effects on fish, particularly in the kidneys, and can affect the endocrine system (Sugawara 2011). Additionally, oxybenzone can cause down-regulation of certain genes in the testes, suggestive of anti-androgenic activity (Blüthgen et al. 2012). On top of that, deformities in the tails of fish embryos can be brought about by exposure to this UV agent. This is particularly alarming, as it can influence the hatching of fish eggs (Balázs et al. 2016) (see Table 5). Oxybenzone can be metabolized to benzophenone-1 in adult fish only, as the enzymes needed for this procedure are not yet active in eleuthero-embryos (Blüthgen et al. 2012).

**Table 5:** Effects of UV filters on marine vertebrates

UV Filter	Effects on Marine Vertebrates	Species Studied	Test Concentration Ranges	Toxicity Threshold	Duration of Study	Reference
OXYBENZONE	Level alterations of VTG	*Danio rerio*	C = 1000 μg/L	na	48 h	Rodríguez-Fuentes et al. 2015
	Anti-androgenic effects to male fish	*Danio rerio*	C = 84 μg/L	na	14 days	Blüthgen et al. 2012
	Tail deformation of hatching embryos	*Danio rerio*	C = 1.10E-01 mM to 4.38E-03 mM	EC_50_ = 5.43E-02 mM = 12.39 mg/L	72 hpf	Balázs et al. 2016
BENZOPHENONE-2	Reduction of spawning rate in female fish	*Pimephales promelas*	C = 1.2 mg/L	na	15 days	Weisbrod et al. 2007
	Cessation of spawning	*Pimephales promelas*	C = 5.0 mg/L, C = 9.7 mg/L	na		Weisbrod et al. 2007
	Suppression of gametogenesis in fish of both sexes	*Pimephales promelas*	C = 1.2 mg/L	na		Weisbrod et al. 2007
EHMC	Enhancement of developmental disorders of the circulatory system	*Danio rerio*	C = 1000 mg/kg	na	48 h	Kaiser et al. 2012
3-BC	Reduction or suppression of spawning rate	*Pimephales promelas*	C = 0.5 μg/L to 285 μg/L	LOEC = 0.5 μg/L	21 days	Kunz et al. 2006
	Histological deformations in testes of male fish	*Pimephales promelas*	C = 0.5 μg/L to 285 μg/L	LOEC = 3 μg/L		Kunz et al. 2006
TiO_2_ (nano)	Gill hyperplasia and swelling of mucocytes	*Oncorhynchus mykiss*	C = 0.1 mg/L, C = 0.5 mg/L, C = 1 mg/L	LOEC = 0.1 mg/L	14 days	Federici et al. 2007
	Changes in superoxide dismutase levels (antioxidant enzyme)	*Cyprinus carpio*	C = 10 mg/L to 200 mg/L	LOEC = 50 mg/L	up to 8 days	Hao et al. 2009
	Changes in catalase and peroxidase levels (antioxidant enzymes)	*Cyprinus carpio*	C = 10 mg/L to 200 mg/L	LOEC = 10 mg/L		Hao et al. 2009
ZnO (nano)	Tissue ulceration in larvae	*Danio rerio*	C = 5 mg/L	na	96 h	Zhu et al. 2008

In the species *Oryzias latipas*, oxybenzone may have an impact on the concentrations of both testosterone and estradiol, two hormones of vital importance regarding reproduction and sexual differentiation. This can be attributed to decrease of production of certain enzymes, particularly aromatase, which is capable of transforming androgens to estrogen. As a result, fish egg production in females is diminished. Benzophenone-1 is also partially at fault for this, along with other products (Coronado et al. 2008, Kim et al. 2014).

In another study it was noted that in zebrafish embryos, benzophenone-4 caused changes in concentration levels of gene products, linked not only to VTG and aromatase synthesis, but also the production of vital enzymes for the process of steroidogenesis (Zucchi et al. 2011a).

Hormonal disruption is not solely caused by benzophenones. Many other UV filters can have a similar impact on marine vertebrates. For example, EHMC can affect hormones in both male and female fish of the species *Pimephales promelas*. In addition to this, it can also diminish the production of spermatocytes and increase the number of mature spermatids in the testes, whereas it decreases the number of oocytes in female ovaries (Christen et al. 2011). In another study, exposure to EHMC (C = 2.2 μg/L) resulted in differential expression of 1096 genes linked to tissue remodeling, wound repair, immune system response, inflammatory response, cell differentiation and cycle. These findings are extremely important, due to the fact that the concentration levels of EHMC in this study are environmentally realistic (Zucchi et al. 2011b).

The UV filter 3-benzylidene camphor (3-BC) can have a negative influence on the reproductive system of fish. It is capable of increasing the transcription rate of the VTG gene, but decrease spawning in *Pimephales promelas*, and if it is present in high concentrations it can cease spawning altogether (dose-dependent response). It can also cause histological abnormalities in the reproductive system comparable to the aforementioned that stem from the UV agent EHMC; in males, spermatids proliferate and accumulate in the tubules. Similarly, in females the number of oogonia is increased (Kunz and Fent 2006, Kunz et al. 2006).

Octocrylene is another UV agent, which is harmful to the reproductive system. A study using zebrafish showed that octocrylene helped decrease the percentage of primary oocytes and increase vitellogenic oocytes in female ovaries (Zhang et al. 2016). It should also be noted that in general, it can affect the expression of certain genes linked to sexual differentiation (Meng et al. 2021). Exposure of the gonads to such an agent can lead to sex reversal (Scholz et al. 2008).

Inorganic UV filters can be toxic to fish due to the metal ions from nanoparticles. The latter can end up in the digestive tract via consumption of the sea water and be detrimental to the respiratory or the digestive system. Additional damage might be present in veins or the liver, where tumor formation may occur (Handy et al. 2008). In a study, manufactured nano-sized TiO_2_ led to a physiological damage on the trout species *Oncorhynchus mykiss*; gill pathologies such as edema and increase of mucus secretion after morphological changes of mucocytes were noted. These results point to respiratory difficulties of the fish, which can play a major role on the integrity of the trout (Federici et al. 2007). TiO_2_ has also been proven to cause oxidative stress, increase of breathing frequency, bewildered swimming and changes in superoxide dismutase and catalase activity (Hao et al. 2009).

Sunscreen agents pose a threat not only to fish, but to dolphins and sea turtles as well. For instance, traces of UV filters have been detected in the bloodstream of the loggerhead turtle *Caretta caretta* and the liver and placenta of the franciscana dolphin *Pontoporia blainvillei* (Cocci et al. 2020, Gago-Ferrero et al. 2013). To elaborate, homosalate, ethyl salicylate, ensulizole and BP-3 were found in loggerhead turtles, the latter in 37% of the individuals (Cocci et al. 2020). Regarding dolphins, octocrylene was detected in 36% of the individuals studied, in concentrations ranging from 89 ng/g lw (lipid weight) to 782 ng/g lw (Gago-Ferrero et al. 2013). In addition to this, the occurrence of benzotriazoles in hammerhead sharks from Japan has also been proven (Nakata et al. 2009).

## Bioaccumulation and biomagnification

Organic UV filters are compounds that have a high octanol/water partition coefficient. As previously mentioned, this translates to poor solubility in water, resulting in bioaccumulation of the pollutants in marine environments and species (Tovar-Sánchez et al. 2020). Apart from this, the phenomenon of biomagnification is referred to as “the increase in contaminant concentration with increasing trophic status of organisms sampled from the same food web” (Drouillard 2008). Biomagnification and bioaccumulation are linked, since the contaminants that accumulate in low-trophic level species are later detected in their predators. Sharks, turtles, dolphins travel large distances and thus do also connect different ecosystems contributing to biomagnification. This phenomenon is the reason that harmful substances are transferred to different ecosystems. The observation of EHMC in cormorants in Switzerland (Fent et al. 2010) is one example. Similarly, accumulation of benzotriazole in marine birds in Japan has been reported (Nakata et al. 2009). Seafood consumption may lead to ingestion of sunscreen agents by humans (Binelli and Provini 2004). As already stated, studies have indicated presence of UV filters in human breast milk, urine and plasma (Janjua et al. 2008, Ngoc et al. 2019). This is worrying, since certain UV agents, such as oxybenzone, can be characterized as hormonal disruptors, and further highlights the toxic effect of biomagnification in humans (Huang et al. 2021).

## Discussion

This study demonstrates the urgent need of selecting ecofriendly and efficient UV filters to be used in sunscreens. The transport of these products to the environment can pose a severe threat on marine biota. It is worth noting the harmful accumulation of sunscreens in many organisms, which may be a gateway for their transfer to higher food levels and completely different ecosystems. The lack of current understanding of the full impacts of UV filters, both in the laboratory and in the environment, represents a significant challenge in interpreting the environmental risk associated with the widespread use of sunscreens.

In recent years, the demand for environmentally friendly and sustainable sunscreen products has increased. In the European Union, UV filters, which are considered as safe during application are listed on the positive list for UV filters – ANNEX VI of the Cosmetics Regulation (EC) No. 1223/2009, so that they can be used in sunscreen formulations. Often, specifically UV filters for use in sunscreens are exempted from chemical registration as they have beneficial aspects to human health (i.e. Japan, Korea, Australia, United States, Canada). In these countries, UV filters have to undergo a specific registration process similar to the European Union, but sometimes even require the higher standards of pharmaceutical registration (United States, Canada, Australia). In the USA, sunscreens are classified Over-The-Counter (OTC) drugs. This means they must comply with all other requirements listed in the FDA’s OTC sunscreen monograph. Individual sunscreen active ingredients are reviewed by FDA and only those that are on FDA’s monograph approved list may be used in sunscreen products marketed in the U.S. (Pavlou et al. 2021). There hasn’t been a single new UV filter approved in the US for the last 10 years. That means, ‘old’ UV filters that may be harmful to biota are still being used instead of ‘new’ more ecofriendly UV filters.

Additionally, the fact that sunscreens can be transferred directly to the aquatic environment via recreational activities, strengthens the necessity of biodegradable sunscreen ingredients. According to the OECD 301 test guidelines, a substance can be labeled as “readily biodegradable” if it fully degrades in a readily biodegradation test using activated sludge from a sewage treatment plant in a 10-day time window, whereas the label “biodegradable” is given when substances degrade within 28 days. However, as mentioned above, implementing environmentally friendly ingredients into sunscreen formulations is quite difficult, especially when human safety and regulations regarding animal testing are taken into consideration (Tovar-Sánchez et al. 2020). It would be worth mentioning that some emphasis should be taken to educate the public on alternatives to applying sunscreens. In particular, clothing, shade-seeking behavior, wearing of sunhats and sunglasses, use of sunscreens on uncovered skin areas and the amount of time spent outdoors, would help to decrease UV filter influx to aquatic environments (Miller et al. 2021).

Seven places all over the world have so far taken regulatory action by banning toxic sunscreen ingredients on their ground. One of the forerunners is the U.S. Virgin Islands which signed a bill into law in July 2019, outlawing all imports, and sales of sunscreens containing the chemicals oxybenzone and octinoxate, which are harmful to the country’s coral reefs. Hawaii, another state in the US, has voted to ban the sale of sun protection products that contain reef damaging chemicals. After scientists conducted research on the effects of Oxybenzone and Octinoxate on reefs, the island of Bonaire, an island municipality of the Netherlands, also unanimously voted to ban the sale of reef-killing sun protection on its shores by 2021. Palau enacted a sunscreen ban. Starting in 2020 businesses could be fined up to 1000 Dollars for selling non-biodegradable sunscreens. Mexican vacations spots, like swimming holes in the Riviera Maya, have already been requesting that their visitors only use mineral and biodegradable sunscreen for some time. Thailand also banned sunscreens with chemicals that damage coral from all of its marine national parks. This is the latest attempt by the Tai government to protect its coral reefs from the tourism industry. The banned sunscreens are those containing oxybenzone, octinoxate, 4-methylbenzylidene camphor (Hutton 2021). These substances are suspected to be endocrine disruptors and to affect the development of corals, even in small quantities.

Education and outreach campaigns, as well as widespread news coverage already raised public awareness about the potential negative effects of sunscreen products on coral reefs. Following the public debate and the implementation of sunscreen bans, consumers are progressively concerned about the potential impacts their sunscreen products may have on the coral environment (Sharifan 2020). Consequently, several cosmetic manufacturers have already acted on these concerns by incorporating claims such as ‘reef safe’ or ‘reef friendly’ into the marketing strategies of their sunscreen products. According to a survey conducted by Levine in Hawaii (Levine 2020), a vast majority of people are willing to purchase sunscreens labeled to be harmless for coral reef environments. There is a tendency towards the use of inorganic UV filters in so-called ‘reef safe’ sunscreens. There is a general belief that inorganic UV filters are, due to their natural origin, safe for the environment. However, whether the use of a substance is being safe for the environment, relies on two basic aspects: (1) the intrinsic hazard profile and (2) the results of an environmental risk assessment (ERA) (Miller et al. 2021).

Sunscreen sustainability is a challenging issue to tackle. In general, sunscreens should be safe for both humans and the environment. When ingredients incorporated are not environmentally classified or labeled according to the Global Harmonized System (GHS), then their expected concentrations in the environment should remain below the Predicted Environmental No-effect Concentrations (PNEC) (Tovar-Sánchez et al. 2020). In more detail, environmental chemical management uses environmental risk assessments and available standardized approaches for effluent-receiving freshwaters where exposure is characterized as a predicted environmental concentration (PEC) and compared with a PNEC. When these are not presented, exposure models are used to derive PECs in proactive and protective ERA, including regulatory ERA frameworks in the United States and the European Union. More specifically, standardized ERA approach recommends firstly employment of conservative assumptions. If negligible risk is identified, the suggestion is that the chemical under investigation is unlikely to cause a risk to the environment and further work is not prioritized. On the other hand, if a risk is identified at the first steps, additional data should be collected in order to identify the risk (Burns et al. 2021).

In conclusion, the UV protection is of paramount importance. On the other hand, there are indications that some sunscreen filters although have been proved safe for human health, may cause damage both in the immediate aquatic environment in which they escape and in the most distant through the phenomenon of biomagnification. Studies related to the stability of sunscreens, their transportability in the aquatic environment in correlation with their physicochemical properties and the possible toxicity of their metabolites on the aquatic organisms, are needed. Particular emphasis should be given on the concentration that some sunscreens may cause damage to the marine ecosystem, as well. We think that a real challenge for researchers is the design of studies focused on the environmental impact of sunscreens and for the cosmetics industry is the subsequent development of safe, efficacious and eco-friendly skin care products.
